# Small chaperons and autophagy protected neurons from necrotic cell death

**DOI:** 10.1038/s41598-017-05995-6

**Published:** 2017-07-18

**Authors:** Ye Lei, Kai Liu, Lin Hou, Lianggong Ding, Yuhong Li, Lei Liu

**Affiliations:** 1 State Key Laboratory of Membrane Biology, School of Life Sciences, Peking University, Beijing, 100871 China; 2 Aging and Disease lab of Xuanwu Hospital and Center of Stroke, Beijing Institute for Brain Disorders, Capital Medical University, Youanmen, Beijing, 100069 China

## Abstract

Neuronal necrosis occurs during early phase of ischemic insult. However, our knowledge of neuronal necrosis is still inadequate. To study the mechanism of neuronal necrosis, we previously established a *Drosophila* genetic model of neuronal necrosis by calcium overloading through expression of a constitutively opened cation channel mutant. Here, we performed further genetic screens and identified a suppressor of neuronal necrosis, *CG17259*, which encodes a seryl-tRNA synthetase. We found that loss-of-function (LOF) *CG17259* activated eIF2α phosphorylation and subsequent up-regulation of chaperons (Hsp26 and Hsp27) and autophagy. Genetically, down-regulation of eIF2α phosphorylation, *Hsp26/Hsp27* or autophagy reduced the protective effect of LOF *CG17259*, indicating they function downstream of CG17259. The protective effect of these protein degradation pathways indicated activation of a toxic protein during neuronal necrosis. Our data indicated that p53 was likely one such protein, because p53 was accumulated in the necrotic neurons and down-regulation of p53 rescued necrosis. In the SH-SY5Y human cells, tunicamycin (TM), a PERK activator, promoted transcription of *hsp27*; and necrosis induced by glutamate could be rescued by TM, associated with reduced p53 accumulation. In an ischemic stroke model in rats, p53 protein was also increased, and TM treatment could reduce the p53 accumulation and brain damage.

## Introduction

As the second most prevalent disease worldwide, brain ischemia causes massive damage to the cells in the brain. However, there is still much progress needs to be made for neuroprotection in clinics. Cell death in brain ischemia is primarily induced by glutamate through the excessive excitation of NMDA receptors, which leads to the dysregulation of calcium homeostasis^1, 2^. In treatments of ischemic stroke, prevention of calcium entry by antagonizing the NMDA receptors has been intensively investigated. However, several NMDA receptor antagonists have failed in clinical trials, likely due to their interference with normal functions of the NMDA receptors^3^. Therefore, identifying key regulators downstream of calcium entry may be desirable for drug targeting.

In response to an insult, both survival and death signals are activated and their balance determines cell fate^4^. For neuronal cell death, different insults may activate either apoptotic or necrotic pathways, and these pathways may positively and negatively crosstalk with each other^5, 6^. For instance, as apoptotic initiators, caspases may have anti-necrotic function through the degradation of the AMPA receptors^7^. Conversely, as a necrotic trigger, calpain can cleave caspase-3^8^ and apoptosis regulatory proteins, such as apoptosis protease-activating factor-1, Bcl-xL, Bax, Bid and p53^5^. Due to this complexity, identifying key targets that regulate both apoptosis and necrosis is important.

Previously, we have generated a calcium-overload-induced necrosis model in *Drosophila* via neuron-specific expression of a constitutively opened cation channel, the glutamate receptor 1 Lurcher mutant (GluR1^Lc^)^9^. Here, we performed genetic screens and identified a new suppressor of neuronal necrosis, CG17259. We found loss-of-function (LOF) *CG17259* induced up-regulate small chaperons (Hsp26 and Hsp27) and autophagy. These degradation pathways might coordinate to degrade p53, which was accumulated in necrotic neurons. In mammalian neurons under a necrotic stress or in a rat stroke model, tunicamycin had protective effect, likely through up-regulation of *hsp27*, similar to the flies. Together, our research provides the genetic evidence for the role of LOF *CG17259* in neuronal necrosis, likely through activation of the eIF2α signaling, small chaperones and autophagy to degrade p53.

## Results

### Genetic screens identified a new suppressor of neuronal necrosis

**Figure 1:**
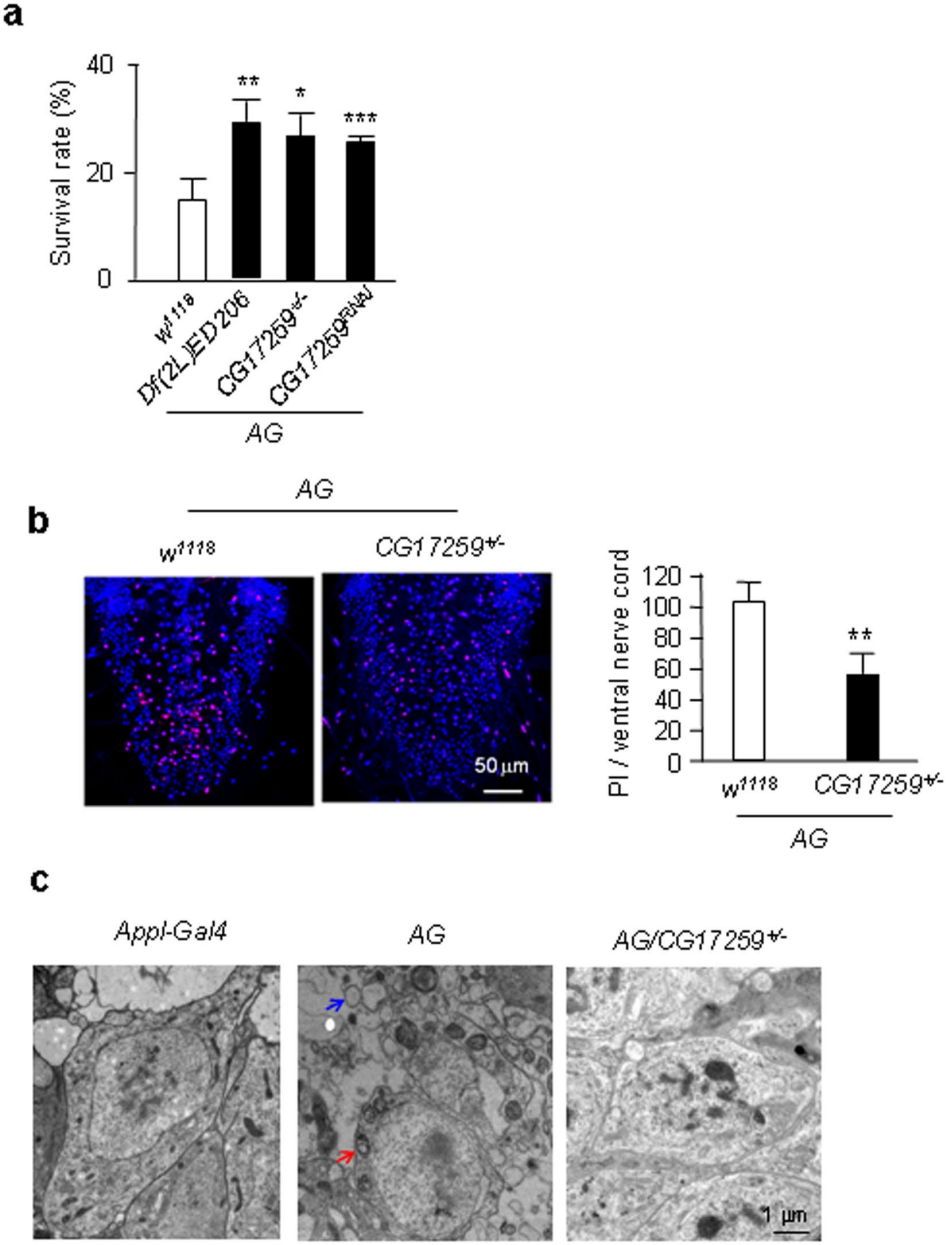
Deficiency screen indentifies a gene as a suppressor of neuronal necrosis. (**a**) Effect of LOF *CG17259* on *AG* fly survival. Df(2 L)ED206 is a deletion mutant; *CG17259* ^+/−^ represents the heterozygous mutant of *CG17259* ^*KG03126*^ and *CG17259* ^*RNAi*^ represents the *UAS-CG17259 RNAi* fly. Trial n = 5. For all *AG* survival experiments, 60–150 flies were tested for each trial. Throughout all bar graphs, error bars are mean + standard deviation (s.d.); white bars represent control; gray bars represent no statistical difference; and black bars represent statistical significant difference from the control (ANOVA for group comparisons followed by post-hoc Tukey test; unpaired *t*-test for comparison of two data sets). Asterisk *for p < 0.05, **for p < 0.01 and ***for p < 0.001. (**b**) Neuronal necrosis in *AG* flies determined by Propidium Iodide (PI) staining. The larval ventral nerve cord was stained with PI, and the statistic result is shown on the bar graph. 10 larvae were examined for each genotype. (**c**) The subcellular characteristic of *AG* fly brain. Representative images from the transmission electron microscope are shown, with the genotype of flies indicated on the micrograph. Red arrow points to a swollen mitochondria; and blue arrow points to a vacuole.

Previously, we have established a genetic model of neuronal necrosis^9^. In this *Drosophila* model, neuronal necrosis was induced by the specific expression of a constitutively open glutamate receptor 1 channel (GluR1^Lc^) in neurons to overload calcium. The fly contains a neuron-specific promoter *A* *ppl-Gal4*, *UAS-* *G* *luR1* ^*Lc*^, and *tub-Gal80* ^*ts*^ (simplified as “*AG*” flies). At 18 °C, the *AG* flies developed normally because Gal4 was suppressed by Gal80^ts^. When placed at 30 °C, the Gal80^ts^ lost its function and permitted the expression of GluR1^Lc^, which resulted in calcium overload and neuronal necrosis^9^. We performed genetic screens using the deficiency lines that cover most of the *Drosophila* genome (the deficiency kit from Bloomington *Drosophila* Stock Center). By screening suppressors of the *AG* fly lethality, we identified nine deficiency lines. Here, the *Df(2 L)ED206* line was further investigated (Fig. 1a). To narrow down the genes in the flanking region of *Df(2 L)ED206*, we tested P-element insertion-mediated mutants in this region and found that loss of *CG17259* (*CG17259* ^*KG03126*^) strongly rescued the *AG* flies (Fig. 1a). By qRT-PCR, we confirmed that the transcript of *CG17259* was reduced by approximately 40% in the heterozygous *CG17259* mutant (*CG17259* ^+/−^) (Fig. S1). Because the homozygous mutant of *CG17259* was embryonic lethal, we also tested a *CG17259* RNAi line (*CG17259* ^*RNAi*^), its effect on the transcript of *CG17259* was confirmed (Fig. S1). The *CG17259* ^*RNAi*^ rescued *AG* lethality (Fig. 1a). At the cellular level, loss of *CG17259* (*CG17259* ^+/−^) reduced neuronal necrosis in the larval ventral nerve cord, as inferred from propidium iodide (PI) staining (Fig. 1b). At the subcellular level, neurons from the brain of adult *AG* flies showed mitochondrial swollen (Fig. 1c red arrow) and vacuole formation (Fig. 1c blue arrow), these are typical features of necrosis^10^. While, *CG17259* ^+/−^ showed rescue effect in the *AG* fly brains (Fig. 1c). Together, these results suggest that CG17259 is a suppressor of neuronal necrosis.

### *CG17259* induced the eIF2α branch of unfolded protein response (UPR)

**Figure 2:**
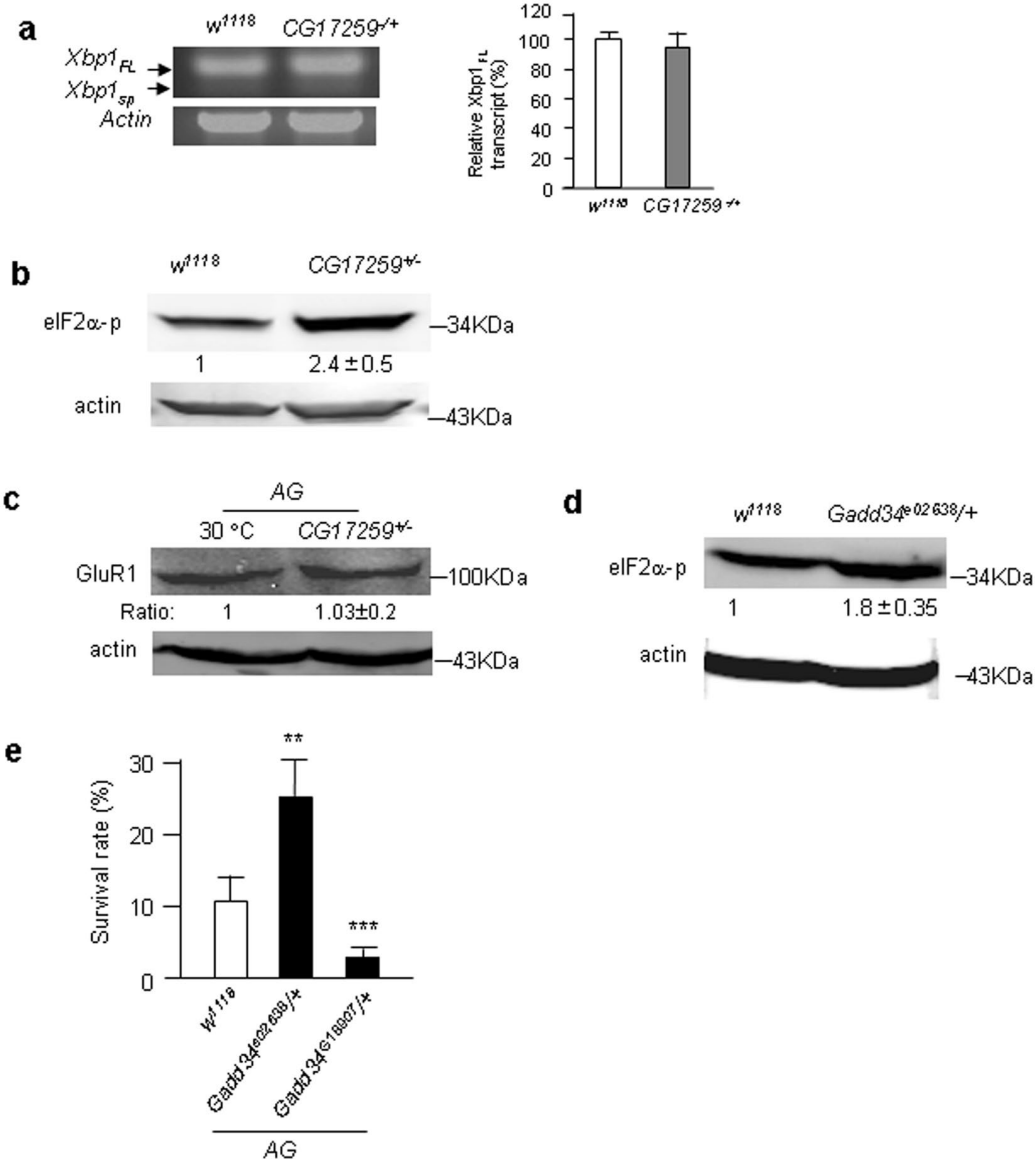
Loss of *CG17259* induced eIF2α phosphorylation. (**a**) The quantitative RT-PCR for *Xbp1*_*FL*_ is shown. The DNA gel with migrations of *Xbp1*_*FL*_ and *Xbp1*_*sp*_ are indicated. *Xbp1*_*sp*_ is not detected. Trial n = 8. (**b**) The Western blot shows the level of phosphorylated form of eIF2α (eIF2α-p) in the *CG17259* mutant flies. The eIF2α-p antibody recognizes the S51 phosphorylation site of eIF2a. Actin is shown as the protein loading control. Trial n = 3. The full length gels of all Western blots were shown at the end of the supplementary information. (**c**) The Western blot shows the level of GluR1^Lc^ in the genotype of flies indicated. Actin is shown as the protein loading control. Trial n = 3. (**d**) The Western blot shows the level of eIF2α-p in the *Gadd34* mutant flies. Actin is shown as the protein loading control. Trial n = 3. (**e**) Survival rate of *AG* flies was shown with the genotype of flies indicated. Trial n = 4.

*CG17259* encodes a seryl-tRNA synthetase, an essential enzyme, which catalyzes the ligation of serine to its cognate tRNA, and thereby affects the fundamental building blocks of protein synthesis^11^. In this respect, *CG17259* ^+/−^ may increase errors in protein synthesis and induce UPR and ER stress. On the ER membrane, three distinct receptors are responsible to UPR, including inositol-requiring enzyme 1 (IRE1), activating transcription factor 6 (ATF6) and double-stranded RNA-activated protein kinase (PKR)-like ER kinase (PERK)^12^. The IRE1 branch can be quantified by increase of a spliced form of *Xbp1* mRNA (*Xbp1*_*sp*_)^13^. We found that full length Xbp1 (*Xbp1*_*FL*_) was unaltered and the *Xbp1*_*sp*_ was undetectable in the *CG17259* ^+/−^ flies (Fig. 2a), suggesting the IRE1 branch was not activated. Next, we tested the PERK branch, which had been shown to be independent from ER stress and can be detected by phosphorylation of eukaryotic translation initiation factor 2 alpha (eIF2α)^14, 15^. Indeed, phosphorylated eIF2α level was increased in the *CG17259* ^+/−^ flies (Fig. 2b). If ER stress was induced in the *AG* flies, protein synthesis of GluR1^Lc^ might be reduced, however, the level of GluR1^Lc^ was unaltered (Fig. 2c). In addition, severe ER stress may induce apoptosis through the activation of ASK1^16^, which, in turn, activates TRAF2-dependent c-Jun N-terminal kinase (JNK) signaling^17^. Using an *in vivo* reporter of JNK activation in *Drosophila* ^18^, the JNK signaling was not activated (Fig. S2); and no cell death was detected in the larval eye discs of *CG17259* ^+/−^ flies (Fig. S3). These results together suggest that no severe ER stress was induced in the *CG17259* ^+/−^ flies. To test the functional importance of eIF2α phosphorylation, we studied GADD34, a regulatory subunit of protein phosphatase 1 (PP1) that dephosphorylates eIF2α^19, 20^. The result showed that the loss of *Gadd34* (*Gadd34* ^*e02638*^) increased eIF2α phosphorylation and suppressed the lethality of *AG* flies (Fig. 2d and e). On the other hand, GOF of *Gadd34* (*Gadd34* ^*G18907*^) had the opposite effect (Fig. 2f). Together, these results suggest that loss of *CG17259* is likely to induce the PERK/ eIF2α signaling branch to suppress neuronal necrosis in the *AG* flies.

### *CG17259* upregulated small chaperones (Hsp26 and Hsp27) and autophagy

**Figure 3:**
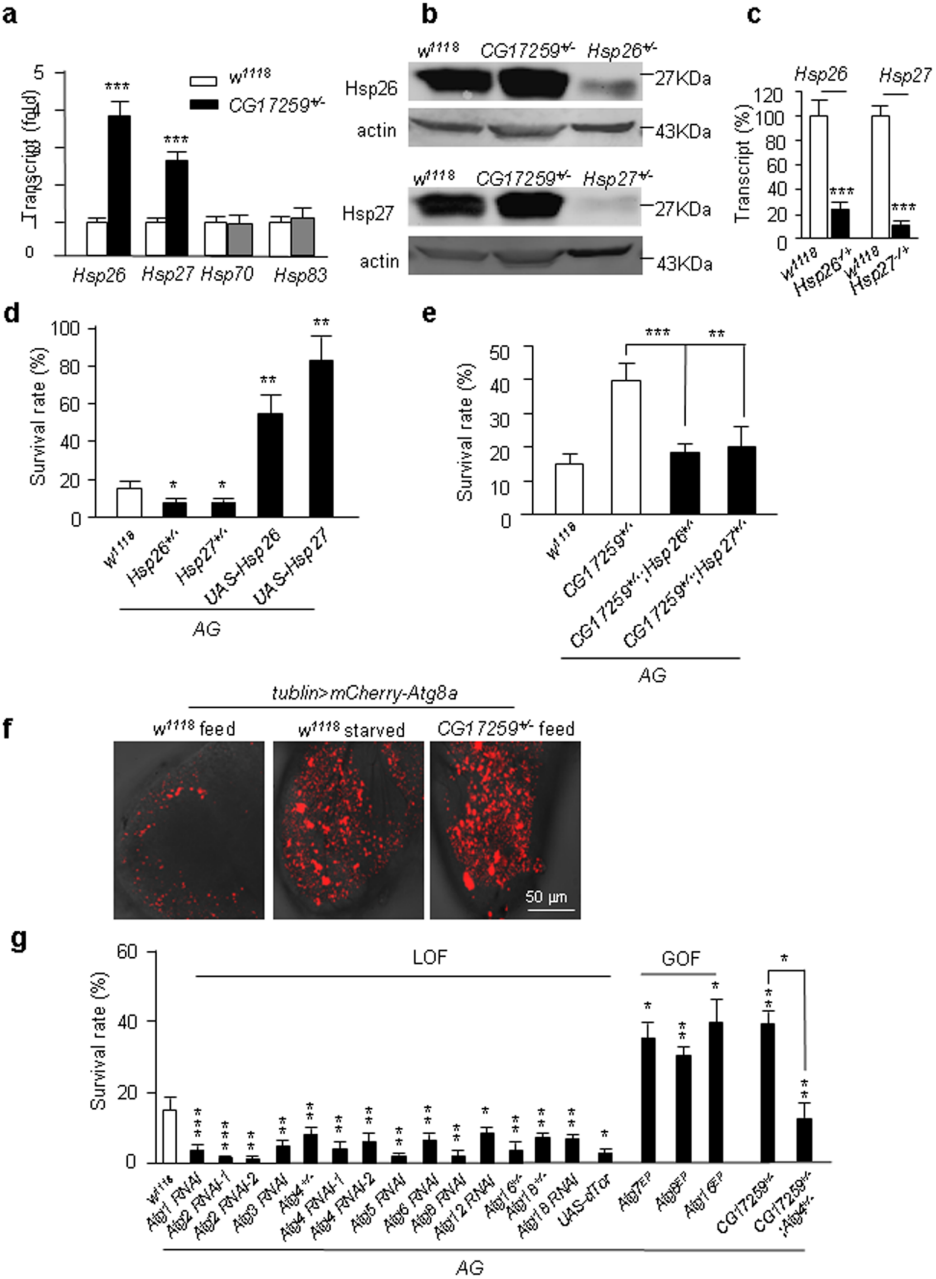
Characterization of the small chaperones and autophagy. (**a**) Transcription level of *Hsp26* and *Hsp27* in the *CG17259* mutant detected by qRT-PCR. The RNAs were collected from the fly heads. The relative mRNA level of *Hsp26*, *Hsp27*, *Hsp70* and *Hsp83* in the wild type fly (*w* ^*1118*^) was defined as 1. The means ± SD of relative transcripts. Trial n = 3. (**b**) The Western blot to detect the protein level of Hsp26 and Hsp27 in the *CG17259* mutant flies. The heterozygous mutants of *hsp26* and *hsp27* serve as controls to indicate the specificity of the antibodies. Trial n = 3. (**c**) qPCR for *Hsp26* and *Hsp27*. The heterozygous mutant of Hsp26^−/+^ and Hsp27^−/+^ lines contain a third chromosome balancer (short bristle) and they are homozygous lethal. The relative levels of transcript of *Hsp26* and *Hsp27* to the wild type (*w* ^*1118*^) are shown. Trial n = 3. (**d**) Survival of *AG* flies under the genetic background indicated. Trial n = 6. (**e**) Survival of *AG* flies under the genetic background indicated. Trial n = 5. (**f**) Autophagy activation determined by the *in vivo* reporter (*mCherry-Atg8a*, the red channel). In the fed condition, the fat bodies of wild type (*w* ^*1118*^) showed minimum activation of autophagy, but autophagy was activated under the starvation condition. This response serves as a positive control for autophagy activation. In the *CG17259* mutant flies, autophagy was activated under the fed condition. Trial n = 2. (**g**) Survival of *AG* flies under different autophagy mutant backgrounds. The RNAi and mutant of the *Atg* genes (as indicated as LOF) all showed enhancing effect on lethality of *AG* flies; whereas up-regulation of several *Atg* genes (GOF) all showed a rescue effect on lethality of *AG* flies. Trial n = 5.

Phosphorylation of eIF2α may activate transcription of chaperones, which help to degrade misfolded proteins in proteasome or lysosome^21^. Indeed, we found that mRNA levels of *Hsp26* and *Hsp27* were elevated; while larger chaperons such as Hsp70 and Hsp83 were unchanged (Fig. 3a). To confirm the increase of Hsp26/Hsp27 at protein level, we generated rabbit polyclonal antibodies and found that both Hsp26 and Hsp27 protein levels were increased in *CG17259* ^+/−^ flies (Fig. 3b). The heterozygous mutant of *Hsp26* (*Hsp26* ^−/+^) and Hsp27 (*Hsp27* ^−/+^) were shown. These protein levels in the heterozygous mutant of *Hsp26* ^−/+^ and *Hsp27* ^−/+^ were much lower in the than in the wild type (*w* ^*1118*^) flies (Fig. 3b), and the transcription levels of Hsp26/Hsp27 in the heterozygous mutant of Hsp26 and Hsp27 were equally reduced (Fig. 3c). These results suggested that the antibodies were specific to detect the Hsp26 and Hsp27 proteins. Moreover, the immunostaining data showed that Hsp26 and Hsp27 were primarily localized in the cytosol/mitochondria and nucleus, respectively (Fig. S4). Functionally, GOF of either protein rescued *AG* fly lethality. In contrast, the LOF of the proteins increased lethality (Fig. 3d). These results indicate that Hsp26 and Hsp27 play a protective role against neuronal necrosis. To test whether Hsp26 and Hsp27 acted downstream of *CG17259*, we generated double-heterozygous mutants, *CG17259* ^+/−^; *Hsp26* ^+/−^ and *CG17259* ^+/−^; *Hsp27* ^+/−^. The results showed that the protective effect of *CG17259* ^+/−^ against *AG* lethality was abolished in flies with either the *Hsp26* ^+/−^ or *Hsp27* ^+/−^ mutant background (Fig. 3e), suggesting Hsp26 and Hsp27 function downstream of CG17259.

Downstream of eIF2α signaling may induce autophagy in various organisms, including *Drosophila* ^12, 22^. It has been reported that autophagy is activated in the *CG17259* ^+/−^ flies ^22^. To replicate this result, we used an *in vivo* reporter of autophagy, mCherry-Atg8a, and observed that its fluorescent puncta were increased in *CG17259* ^+/−^ flies (Fig. 3f). Similarly, this autophagy activation was detectable by a lysotracker staining (Fig. S5). Because role of autophagy on neuronal necrosis is unclear, we focused on the functional study in the *AG* flies. The result showed that targeting genes in the autophagy pathway by RNAi enhanced *AG* fly lethality; and inhibition of Atg1 activity by GOF of *dTor* ^23, 24^ had a similar effect (Fig. 3g). In contrast, GOF of several *Atg* genes rescued fly lethality (Fig. 3g). In addition, the protective effect of *CG17259* ^+/−^ was abolished under the LOF background of *Atg4* (Fig. 3g), suggesting CG17259 functions upstream of autophagy. These results together suggest that autophagy functions downstream of *CG17259* ^+/−^, and it is necessary for the protective role of LOF *CG17259* against neuronal necrosis in *Drosophila*.

### Down regulation of P53 reduced neuronal necrosis in *Drosophila*

**Figure 4:**
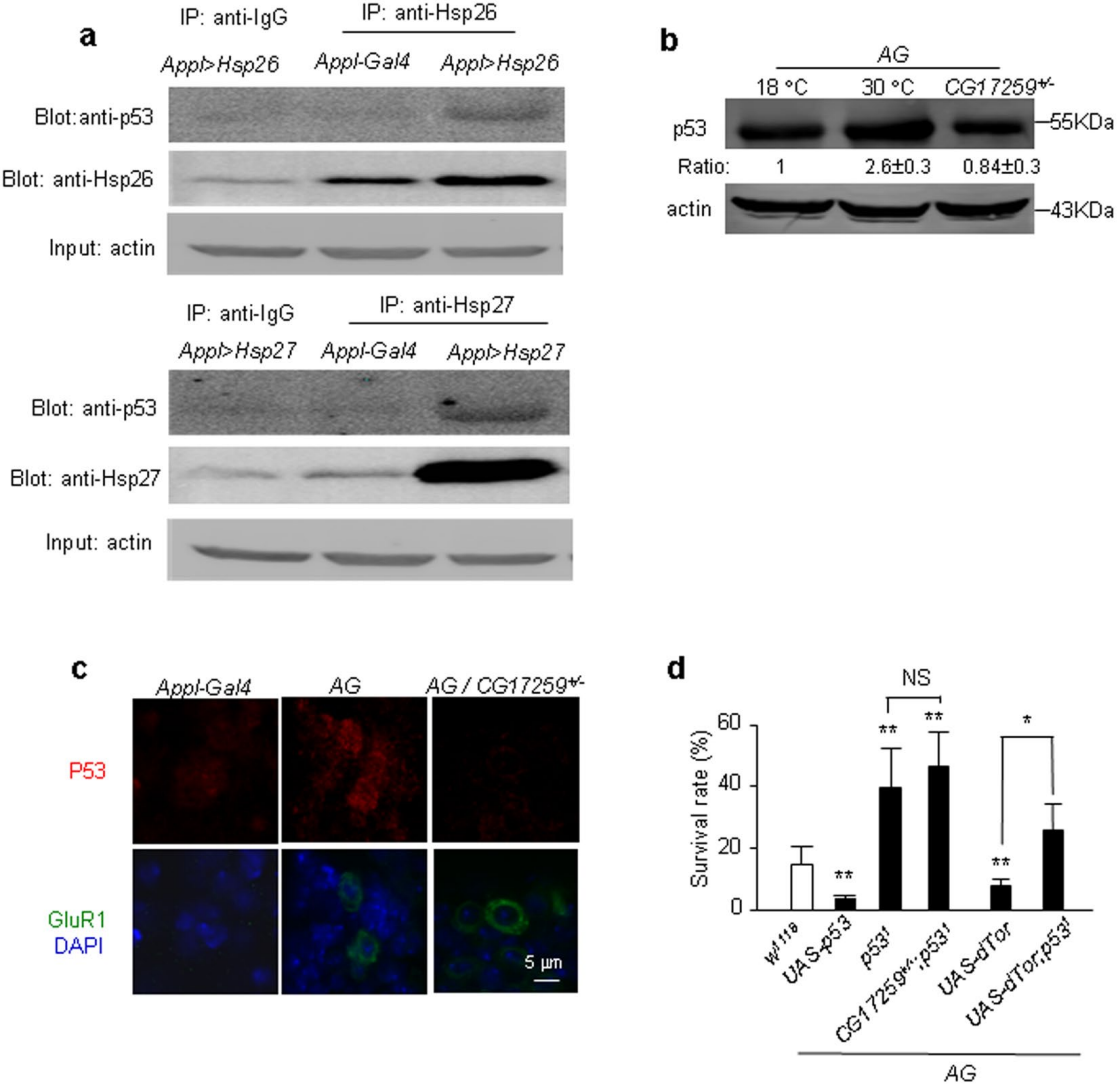
Characterization of p53 in the *AG* flies. (**a**) Co-immunoprecipitation of Hsp26/Hsp27 with p53. The homogenized fly heads were immunoprecipitated with anti-Hsp26 or anti-Hsp27 antibodies and probed with anti-Hsp26, anti-Hsp27, anti-p53 antibody, with anti-IgG as a control. The input of actin is the protein loading control. The genotypes of flies are indicated on the image. Trial n = 2. (**b**) Accumulation of p53 in the *AG* adult flies. The control (*AG* flies at 18 °C) p53 protein level is set as 1, and the indicated genotype flies relative to the control are shown. Trial n = 3. (**c**) Accumulation of p53 in the larval *AG* brain. The images show immunostaining with anti-p53 and anti-GluR1 antibodies. The nuclei are stained with DAPI. The result showed that the 53 protein (the red channel) was lower in the control (*Appl-Gal4*) ventral nerve cord of larval flies; whereas p53 protein level was higher in the *AG* flies, especially in neurons with higher GluR1 expression (the green channel). Trial n = 2. (**d**) Effect of p53 on *AG* fly survival. The survival rate of indicated genotype of flies is shown. Trial n = 4.

The protective effect of small chaperons and autophagy suggests accumulation of misfolded proteins or damaged organelles in neuronal necrosis. To test whether massive amounts of misfolded proteins were generated in the *AG* flies, we preformed immunohistochemical staining and Western blotting to detect the global level of ubiquitinated proteins. Surprisingly, we observed no elevation of ubiquitinated proteins in *AG* fly heads (Figs S6 and S7). This result indicates that a specific protein may be accumulated in the necrotic neurons. Of note, phosphorylation of eIF2α inhibits p53 accumulation and apoptosis in mouse fibrosarcoma cells^25^. Moreover, by searching the database of protein interactions in *Drosophila*, both Hsp26 and Hsp27 interact directly with p53^26, 27^. To confirm these interactions, we conducted co-immunoprecipitation (co-IP) using the Hsp26 and Hsp27 antibodies. Because the wild type (*Appl-Gal4*) flies had very low level of p53, we were unable to detect p53 protein after IP with the anti-Hsp26 or anti-hsp27 (Fig. 4a). However, in the flies with neuron-specific overexpression of *Hsp26* and *Hsp27* (*Appl* > *Hsp26* and *Appl* > *Hsp27*), both Hsp26 and Hsp27 antibodies could pull down p53 (Fig. 4a). This result suggests that Hsp26/Hsp27 may bind with p53.

In the *AG* fly brains, the p53 protein was increased after induction of neuronal necrosis (Fig. 4b); and p53 protein tended to accumulate in neurons with higher GluR1^Lc^ expression in the larval ventral nerve cord (Fig. 4c). These results suggest that p53 protein level was altered in the necrotic neurons.

Functionally, overexpression of *p53* enhanced the *AG* fly lethality and LOF *p53* (p53^1^ is a LOF mutant of *p53*) rescued the *AG* fly (Fig. 4d). The double mutants of *CG17259* and *p53* showed no additive effect (Fig. 4d), suggesting they may function in the same pathway. Whereas *p53* ^*1*^ reduced the lethality of *AG/dT*or flies (Fig. 4d), indicating that inhibition of autophagy enhanced the p53 toxicity. This result is consistent with a report suggesting that degradation of p53 depends on the autophagy pathway in cancer cells^28^. Together, these results suggest that p53 may play a functional role in neuronal necrosis.

### Down regulation of p53 reduced necrosis in mammalian neurons

**Figure 5:**
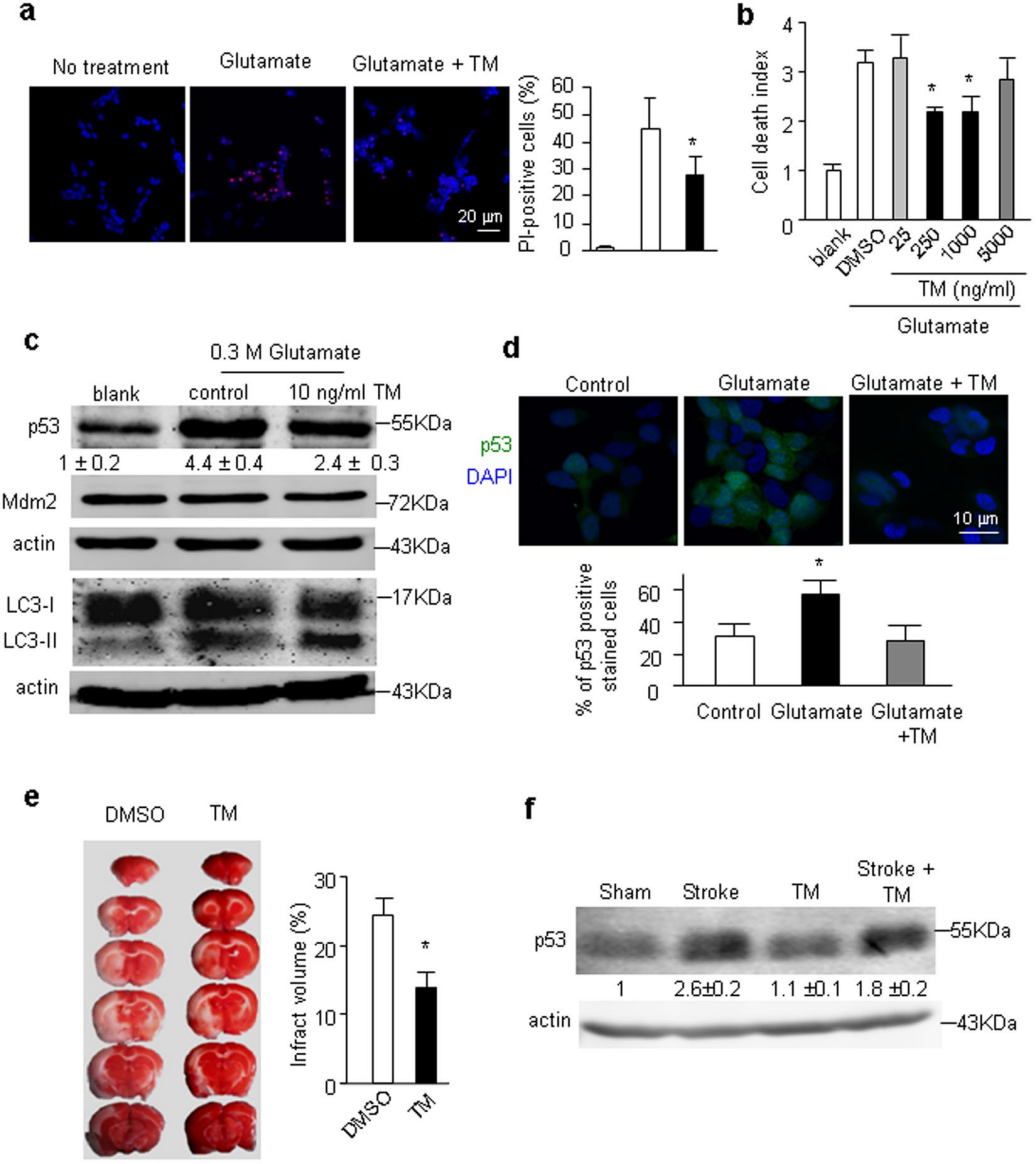
Effect of ER stress on neuronal necrosis and stroke. (**a**) Effect of TM on glutamate-induced cell death in SH-SY5Y cells. The cell death was determined by PI staining. The statistic result is shown on the bar graph. Trial n = 4. (**b**) Effect of TM on glutamate-induced cell death in SH-SY5Y cells. The cell death was determined by the ATP assay. Trial n = 4. (**c**) Protein level of p53 and the active state of autophagy. The result showed that TM treatment reduced p53 accumulation upon glutamate treatment and autophagy was activated as shown by the increased LC3-II level. The Mdm2 protein level shows no change. Trail n = 3. (**d**) Immunofluorescent staining of p53 in SH-SY5Y cells. DAPI labels DNA. The bar graph shows the percentages of p53 positive cells from 3 independent experiments. (**e**) Effect of TM (dissolved in DMSO) on the rat MCAO model. Injection of DMSO is the sham control. The white areas of TTC-stained images show the levels of brain damage. The quantitative data is shown in the bar graph. Five rats were tested for each condition. (**f**) Protein level of p53 detected by Western blot under conditions indicated. Trial n = 3.

To test the effect of activation PERK/eIF2α signaling in mammalian neurons, mouse cortical neurons were pretreated with tunicamycin (TM), an inhibitor of N-linked glycosylation, known to activate the PERK/eIF2α signaling^29^. In primary neuron cultures, TM treatment showed a protective effect against necrosis induced by glutamate (Fig. S8). However, p53 protein level was too low to quantify by Western blot analysis in the primary neuron cultures. Therefore, we turned to SH-SY5Y cells, a human neuroblastoma cell line. In these cells, p53 protein was detectable, and glutamate treatment resulted in an increase of p53 protein (Fig. 5a and b). Importantly, TM pretreatment resulted in a transcriptional activation of *Hsp27*, which is the homolog of *Drosophila* Hsp26/hsp27 (Fig. S9). Furthermore, TM treatment protected against the glutamate toxicity (Fig. 5a and b), and reduced p53 accumulation (Figs. 5c and d). In addition, TM pretreatment enhanced autophagy because the marker of autophagosomes in mammalian cells, LC3-II^30^, was increased (Fig. 5c). Mdm2 is a key E3 ligase for ubiquitination and proteasomal degradation of p53^31^. However, the Mdm2 protein level was unaltered upon TM treatment (Fig. 5c), suggesting that the main degradation pathway for p53 was likely through autophagy in these cells. Consistent with this notion, enhanced autophagy by rapamycin reduced the level of p53 protein (Fig. S10). On the other hand, inhibition of autophagy by 3-MA resulted in the accumulation of p53 protein (Fig. S11). Together, these results suggest that TM treatment mimics the LOF *CG17259*.

To test the potential protective effect of TM against p53 accumulation *in vivo*, we examined an intraluminal middle cerebral artery occlusion (MCAO) model in rats, in which neuronal necrosis is the predominant form of cell death, especially in the ischemic core^32^. Six hours after intraperitoneal injection of TM (0.4 mg/kg), we performed permanent focal occlusion^32, 33^. The infarction volume was assessed by triphenyl-tetrazolium chloride (TTC) staining in the coronally-sectioned brain slices. The defective brain area was not stained by TTC and had a white color^34^. Our results demonstrated that TM treatment significantly reduced infarct volume (Fig. 5e). Moreover, p53 accumulated after stroke, whereas TM pretreatment reduced the increase in p53 accumulation (Fig. 5f). This result suggests that reducing p53 by eIF2α signaling pathway may be a novel strategy for preconditioning neurons against ischemic insult.

**Figure 6:**
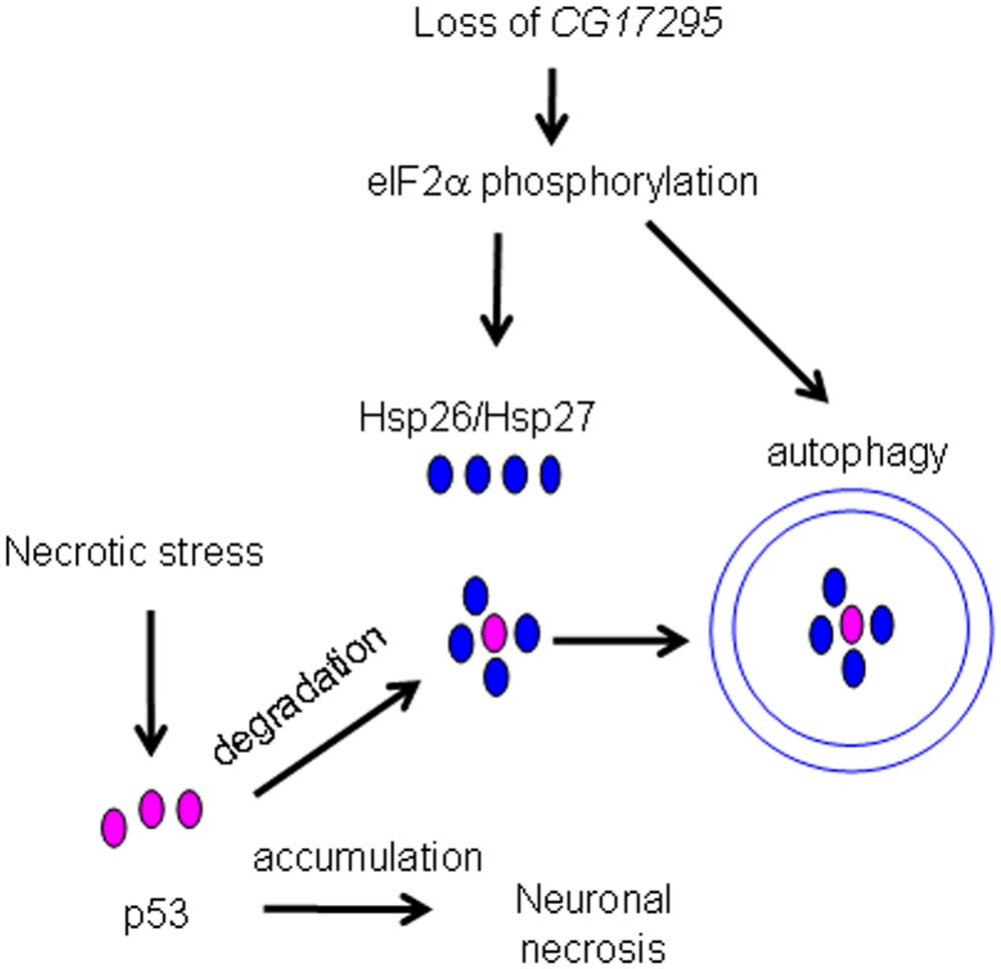
Model of CG17259 function in neuronal necrosis. The model of CG17259 functions against neuronal necrosis. Loss of *CG17259* may induce eIF2α signaling pathway, and further activates the chaperon and autophagy pathways. The chaperons (Hsp26/Hsp27) capture the accumulated p53 generated from necrotic stress and degrade them in the autophagy pathway.

In conclusion, loss of *CG17259* may induce the eIF2α signaling pathway, which further activates the chaperon and autophagy pathways. Necrotic stress results in accumulation of p53, which is recognized and degraded by Hsp26/Hsp27 and the autophagy pathway. A graphic summary is shown (Fig. 6).

## Discussion

### Activation of eIF2α signaling plays an important role in the protection of neurons

By genetic screens using *AG* fly lethality, we identified a novel suppressor of neuronal necrosis, LOF CG17259. CG17259 encodes a seryl-tRNA synthetase and functions in ligation of serine to its cognate tRNA. Therefore, LOF CG17259 may affect protein synthesis and induce cytoplasmic protein folding defects and/or ER stress^35^. ER stress initiates through three distinct sensors in the ER membrane, including PERK, ATF6 and IRE1^12^. Each signaling branch has both overlapping and distinct functions. For example, PERK phosphorylates eIF2α to reduce overall protein translation and promote cell survival^36^. Whereas the IRE1 branch reduces protein synthesis by promoting the degradation of mRNA and activates JNK, which may, in turn, induce apoptosis^37^. Our data demonstrated that the IRE1 branch was not activated in LOF CG17259, because transcription of *Xbp1*_*sp*_ and JNK pathway were not activated. In contrast, and the PERK/eIF2α branch was up-regulated in the LOF CG17259 flies. Consistent with our data, activation of the PERK/eIF2α signaling branch has been implicated in the treatment of various neurodegenerative diseases. For instance, treatment with salubrinal, an inhibitor of eIF2α dephosphorylation, can rescue neurodegeneration in α-synuclein transgenic mice or ischemic stroke in rats^38, 39^. Further, we found that autophagy was activated in LOF CG17259. The coupling of the PERK/eIF2α signaling branch with autophagy has been well documented to protect neurons^40^. Our research is consistent with these results from the literature. Additionally, our research provides an additional mechanism by which the eIF2α signaling pathway affects neuron survival.

### Small chaperones regulate p53 protein abundance

Our results showed that the rescue effect of *CG17259* ^−/+^ was abolished by the mutants of *Hsp26/Hsp27*, and overexpression of Hsp26 or Hsp27 was sufficient to rescue AG flies, suggesting Hsp26/Hsp27 are down stream of LOF *CG17259*. The small chaperones of *Drosophila* Hsp26/Hsp27 are likely to have a similar function to that of mammalian Hsp27, which is known to protect neurons under various pathological conditions, including ischemic stroke^41^. The protective mechanisms of Hsp27 may involve the suppression of the formation of actin aggregates, activation of the NF-κB pathway, or direct inhibition of components in the apoptotic machinery^42–45^. The mammalian Hsp27 may share the combined function of *Drosophila* Hsp26/Hsp27, because it localizes in both cytosol and nucleus upon phosphorylation; while, it mainly localizes in the nucleus upon dephosphorylation^45^. Our data showed that the *Drosophila* Hsp26 and Hsp27 distributed in cytosol or nucleus, respectively. For functional study, our data suggests that Hsp26/Hsp27 and p53 may function in the same pathway, because the rescue effect of *p53* ^1^ and *CG17259* ^−/+^ was not additive and Hsp26/Hsp27 protein could pull down p53. Although the co-IP data was obtained under the Hsp26/Hsp27 overexpression condition, the interaction between Hsp26/Hsp27 and p53 has been reported by other studies^26, 27^.

### Autophagy regulates neuronal necrosis likely through degradation of p53

The autophagy pathways can be further classified into autophagy (in this text macroautophagy refers to autophagy) and chaperone-mediated autophagy (CMA). Autophagy requires the formation of autophagosomes and the function of *Atg* genes. In contrast, the CMA pathway degrades proteins in lysosomes and does not require *Atg* genes^46^. Our data suggested that autophagy was activated in the LOF CG17259 flies; up-regulation of autophagy rescued the AG lethality and down-regulation of autophagy had the opposite effect. Because LOF p53 rescued the enhancing death effect of LOF autophagy, it is possible that degradation of accumulated p53 was dependent on autophagy in the *AG* flies. Consistent with our data, the increase in the level of p53 protein has been observed in embryonic fibroblasts in *Atg7* ^−/−^ or *Atg5* ^−/−^ mice^47^.

### Accumulation of p53 plays a key role in neuronal necrosis; and p53 may function at the convergent code for both neuronal apoptosis and necrosis

Function of p53 in apoptosis has been well documented^48–50^. Upregulation of p53 has been linked to neuronal cell death in numerous models of injuries and diseases^51^, including excitotoxicity^52^. The absence of p53 protects neurons from a wide variety of toxic insults, including focal ischemia^53^, ionizing radiation^54^ and MPTP-induced neurotoxicity^55^. In response to various types of stress, p53 promotes apoptosis through either transactivation of specific target genes^50^ or transcription-independent pathways^49^. As a transcription factor, p53 upregulates proapoptotic genes, such as Bax, Noxa and PUMA^56^. In addition, p53 can interact with Bcl2 family proteins, such as Bax and Bak, to induce permeabilization of the outer mitochondrial membrane^57, 58^. Whether p53 is involved in neuronal necrosis is unclear. In support of its involvement in necrosis, p53 may physically interact with cyclophilin D (CypD), a component of the mitochondrial permeability transition pores and trigger the opening of the pores and necrosis^59^. In addition, the formation of the p53-CypD complex occurs during brain ischemia/reperfusion insult^59^. Here, we provide the genetic and cell biology evidence indicating that p53 is involved in neuronal necrosis. In SH-SY5Y cells, we showed that p53 was accumulated upon cells treated with glutamate; and this accumulation was prohibited by TM treatment, which enhanced Hsp27 transcription. Similarly, the increased level of p53 in MCAO rat brain was down-regulated by TM treatment. Together, these results indicate conserved function of p53 in neuronal necrosis. In fact, protective effect of TM against neurodegeneration has been widely reported^60–62^. The difference is that our study evaluated potential down-stream function of TM to degrade p53 in neuronal necrosis. How does p53 trigger both apoptosis and necrosis? We propose that mild p53 accumulation likely induces apoptosis, whereas the additional accumulation of p53 promotes necrosis. This hypothesis requires further investigation however.

### Potential clinical applications

The inhibition of p53 transcriptional activity by pifithrin α or its mitochondrial targeting by pifithrin μ protects the brain in rodent models of stroke^63–65^. However, p53 also benefits animal survival under hypoxic conditions^66^. Thus, administration of pifithrins may interfere with the normal function of p53 and thereby produce side effects. An alternative way to target p53 may be to aim to reduce the accumulation of p53. Our research suggests that the promotion of eIF2α signaling may activate endogenous mechanisms (activation of small chaperones and autophagy) to degrade p53.

## Methods

### Fly maintenance and stocks

Flies were raised on standard sucrose/cornmeal medium at constant 18 °C or 25 °C with 12 hours light and 12 hours dark cycle. For *CG17259* RNAi lines, transgenic lines were obtained from the *w* ^*1118*^ background using the SympUAST-w vector^67^ to target −100 to −1 in the 5′-UTR from the translational start site of *CG17259*. The other RNAi (TRiP) lines were obtained from the Tsinghua *Drosophila* stock center (Beijing, China). All other fly lines were obtained from The Bloomington *Drosophila* Stock Center (Bloomington, IN, USA).

### Genetic screening

The *AG* flies were crossed with deficient lines or point mutations or P-element-based mutations or RNAi lines obtained from the Bloomington *Drosophila* Stock Center, and raised at 18°C. The 3-day-old progeny flies were incubated at 30°C for 13 h, then transferred back to 18°C and after 2 days, their survival rate was recorded.

### Antibodies

The following antibodies were used in this study (W: Western blot; IF: immunofluorescence; IP: immunoprecipitation): p53 (sc-6243,W), actin (transgen, HC-201, W); p53 (DSHB, 25F4C, IP and W); eIF2αph (ab32157, W); Hsp26 (our own lab, Rabbit polyclonal, IF, IP and W); Hsp27(our own lab, Rabbit polyclonal, IF, IP and W); p53 (CST, 1C12#2524, W); LC3 (NB100-2331,W); Mdm2(GTX110608,W); p53(sc-126, W); ubiquitin (13-1600, Invitrogen, IF and W).

### Transmission Electron Microscope

Heads was collected and fixed overnight on ice in 2.5% glutaraldehyde. The heads were then washed for 3 × 10 min in PB and postfixed on ice for 4 h in 1% osmium tetroxide. The samples were then washed at room temperature 3 × 10 min in H_2_O and dehydrated for 1 × 10 min in 25, 50, 70, 80, 90% acetone and 2 × 10 min in 100% acetone. The heads were then incubated for 3 × 5 min in propylene oxide and incubated overnight in a 1:1 mixture of propylene oxide and Spurr’s medium. The heads were then immersed in 100% Spurr’s for 2 × 2 h and baked in moulds at 60 °C for 24 h.

### PCR primer sets to detect Xbp1_FL_ and Xbp1_sp_

*Xbp1 full* Forward: GCAGGCGCTGAGGGCTGTGC

Reverse: GGACACACAGTCGTCAGCGCGTCT

*Xbp1 splice* Forward: GCAGGCGCTGAGGGCTGTGC

Reverse: AGACGCGCTGACGACTGTGTGTCC.

### qRT-PCR

Total RNA was extracted by Trizol reagent (Invitrogen) followed by DNase I treatment using the manufacturer’s standard protocol, then the purity and integrity of total RNA was determined by 1% agarose gel electrophoresis. The concentration of total RNA was measured by Nanodrop. Five μg mRNA was reverse-transcribed into a cDNA library by oligo-dT primer using Revert Aid First Strand cDNA Synthesis Kit (Thermo scientific) based on the manufacturer’s instructions. For qPCR, the final volume of the q-PCR reaction was 25 μl using a Platinum SYBRGreen qPCR SuperMix-UDG Kit (Invitrogen) containing 1μl diluted cDNA sample (1:3). The qPCR was performed in triplicate using the 7500 real time PCR system (ABI). The quantification of target gene was conducted by ΔΔCt method^68^.

qPCR primer sets for *Drosophila* genes:

*hsp26* Forward: GCTTTCGCTTGTGGATGAACT

Reverse: CCCAGTCCAAGCTCGTAGATG

*hsp27* Forward: GGACGGCCATGGAATGATC

Reverse: GCCCTTGGGCAGGGTATACT

*hsp70-4* Forward: CCAACCAGCTGGCTGACAA

Reverse: GCACACACCCTCCAGTTCCT

*hsp83* Forward: CCGCAATCCCGATGATATCT

Reverse: GTCGTTGGTCAGGGATTTGTAGA

*CG17259* Forward: TGACCTCCCCACACGACAA

Reverse: CCGCATTCCCGATCATCTC

*GAPDH* Forward: CGCAGCGCCATTCTCCTA

Reverse: GACTGCCGCTTTTTCCTTTTC

qPCR primer sets for human gene:

*Hsp27* Forward: TGACCTCCCCACACGACAA

Reverse: CCGCATTCCCGATCATCTC.

### Immunoprecipitation

The fly heads were homogenized and lysed in the lysis buffer (20 mM Tris-HCl, pH7.5, 100 mM NaCl, 0.5 mM EDTA, pH8.0, 0.5% NP40 and ‘Complete’ Protease Inhibitor Cocktail (Roche)). After centrifugation at 13,000 g for 10 min at 4 °C, the supernatant was incubated with Normal IgG antibody (mouse or rabbit, Invitrogen/Life Technologies) and protein A/G-agarose beads at 4 °C for 2 h. After centrifugation at 13,000 g for 10 min at 4 °C, the supernatant was incubated with the requisite antibody and protein A/G-agarose beads at 4 °C overnight. After washing with washing buffer (20 mM Tris-HCl, pH7.5, 500 mM NaCl, 0.5 mM EDTA, pH8.0, 0.5% NP40) 6 times, the agarose beads were boiled in 1 × SDS loading buffer.

### Mammalian cell cultures

SH-SY5Y cells (purchased from Concorde basic medical institute, Beijing, China) were cultured in DMEM supplemented with 15% (v/v) fetal bovine serum and 1%(v/v) penicillin/streptomycin in a 37 °C incubator with 5% CO_2_. We tested for contamination every two months. Different concentrations of TM were used to pretreat these cells. Twenty-four hours later, glutamate (0.3 M) was applied to trigger PNN for four hours. Cell death was quantified by the ATP assay.

For neuron culture, cortical neurons from 18 day-after-fertilization embryos of mice were collected and cultured following a published protocol^69^. After 8–11 days *in vitro* culture, the neurons were treated with 200 μM glutamate for 4 h. Cell death was quantified by the LDH assay.

### Rat ischemia models

All experiments were approved by the Institutional Animal Care and Use Committee, Peking University; and these experiments were performed in accordance with the relevant guidelines and regulations by the university. For the rat MCAO model, adult male Sprague-Dawley rats (250–270 g, Vital River, Beijing), were housed with a 12 h light and 12 h dark cycle. The number of rats per cage was 3 before surgery and they were raised separately after surgery. A permanent MCAO method was used as described previously^70^, with the suture-insertion through the left external carotid artery of anesthetized rats. A laser Doppler blood flow monitor was used to ensure successful occlusion (>80% drop from pre-stroke baseline). TM was injected, intraperitoneally, 6 h before the ischemia surgery. The infarct volume was determined 6 h later. The body temperature was maintained with a heated pad, and the infarct volumes were determined by TTC staining (2%, at 37 °C for 30 minutes) and analyzed using the Image J software.

### Statistical analyses

One-way ANOVA test was used for group comparison, with Tukey’s post hoc test. Student-*t* test with 2-tailed pairing was used to compare two data sets. p < 0.05 is considered statically significant.

## Electronic supplementary material

Supplementary information
