# Supplementary material for: Small chaperons and autophagy protected neurons from necrotic cell death
Source: Sci Rep. 2017 Jul 18;7:5650. doi: 10.1038/s41598-017-05995-6 (PMC5515951; doi:10.1038/s41598-017-05995-6)
Supplement: Supplementary file 1 — Supplementary information [file 41598_2017_5995_MOESM1_ESM.pdf]

Supplementary Information

Title:

Small chaperons and autophagy protected neurons from necrotic cell death

Running title:

Role of small chaperons on neuronal necrosis

Ye Lei[1,2], Kai Liu[1], Lin Hou[1,2], Lianggong Ding[1,2], Yuhong Li[2] and Lei Liu[2]

[1]State Key Laboratory of Membrane Biology, School of Life Sciences, Peking University, Beijing, 100871, China

[2] Aging and Disease lab of Xuanwu Hospital and Center of Stroke, Beijing Institute for Brain Disorders, Capital Medical University, Youanmen, Beijing, 100069, China

Correspondence:

leiliu@ccmu.edu.cn

Supplementary Figure Legends

Figure S1. Effect of *CG17259* mutant and RNAi on *CG17259* transcript. The *CG17259* heterozygous mutant (*CG17259*$^{+/-}$) and the RNAi line reduced the *CG17259* transcripts. Error bars are mean + SEM. Trial n = 3.

Figure S2. Characterization of the activity of the JNK signaling pathway. Larval eye discs were stained with LacZ to detect the JNK signal. JNK reporter line (*puc-lacZ*) is shown as a positive control (green).

Figure S3. Characterization of cell death in the *CG17259* mutant using AO staining of the larval eye disc. *GMR-Eiger* is used as a positive control. The red staining highlights the dead cells.

Figure S4. **Subcellular localization of Hsp26 and Hsp27**

Immunostaining with anti-Hsp26 and anti-Hsp27 in the fly larval brain. DAPI labels the DNA. The result shows that Hsp26 localizes mainly in the cytosol; and Hsp27 localizes mainly in the nucleus.

Figure S5. Autophagy detected by lysotracker staining. Compared to the larval brain of the wild type fly (w^{1118}), the lysotracker staining is increased in the heterozygous *CG17259* mutant.

Figure S6. Characterization of ubiquitin level in the *AG* fly brain by immunofluorescent staining. The *AG* adult brains were stained with anti-ubiquitin antibody. The result shows that the ubiquitinated protein level is not increased under necrotic stress (*AG* flies at 29 °C for 18 hours).

Figure S7. Western blotting reveals the levels of ubiquitin in the *AG* fly brains. The equal amount of protein abstracts were subjected to Western blotting with

anti-ubiquitin antibody.

Figure S8. Effect of TM on glutamate-induced neuronal necrosis in mouse cortical neuron cell cultures. The concentration of TM used is indicated. The cell death index was determined by the LDH release assay. Trial n = 3.

Figure S9. Effect of activation of autophagy on p53 degradation. The SH-SY5Y cells were treated with rapamycin for 24 hours; then, treated with glutamate (0.3 M) for 4 hours. Proteins from the cells were collected and subjected to Western blot. The result showed that pretreatment with rapamycin reduced p53 protein level in glutamate-induced necrosis. Trial n = 3.

Figure S10. Effect of suppression of autophagy on p53 accumulation. 3-MA is an inhibitor of autophagy. The same experimental procedure is used as **Figure. S8**. The result showed that pretreatment of 3-MA increased p53 protein level upon glutamate-induced necrosis. Trial n = 3.

Figure S11. Effect of TM (10 ng/ml) on the Hsp27 transcript level in the SH-SY5Y cells. The qRT-PCR result showed that the transcripts of Hsp27 was increased upon TM treatment. Trial n = 3.

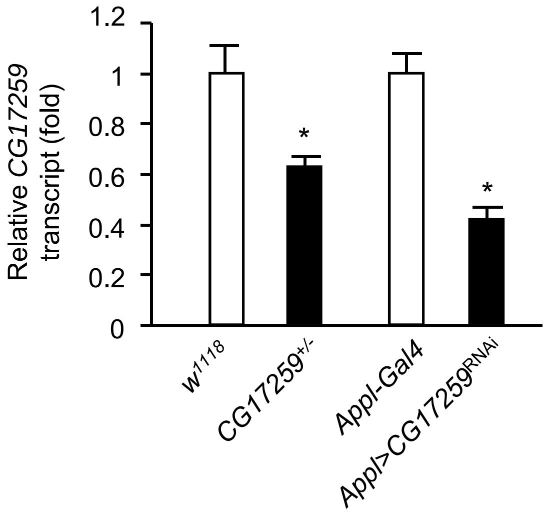

Supplementary Figure 1

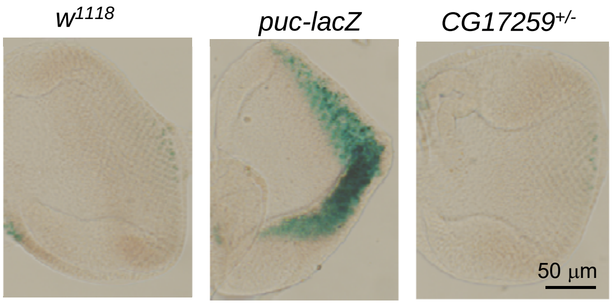

LacZ staining

Supplementary Figure 2

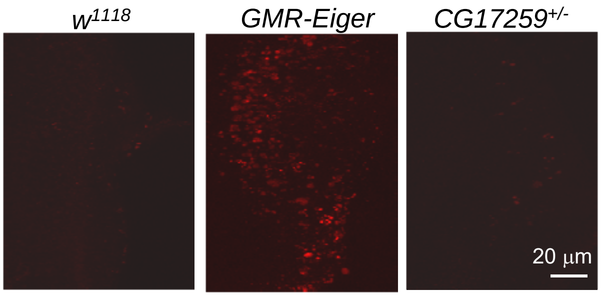

AO staining

Supplementary Figure 3

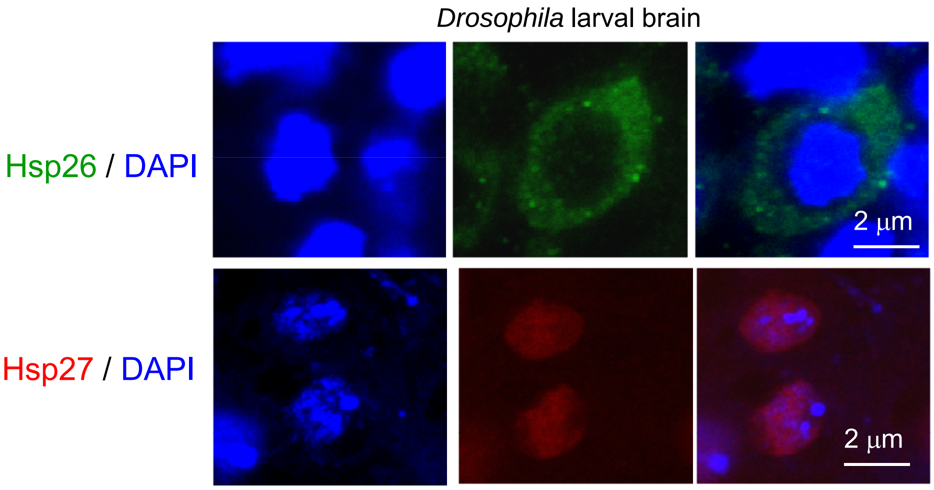

Supplementary Figure 4

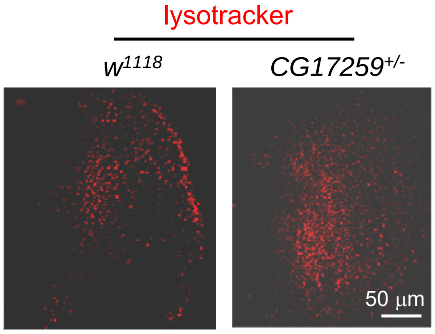

Supplementary Figure 5

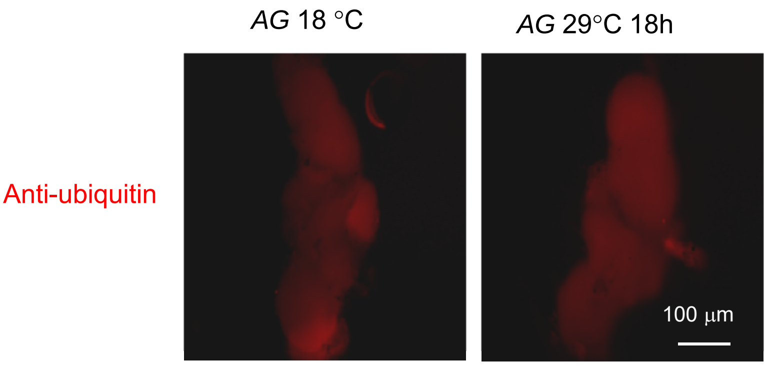

Supplementary Figure 6

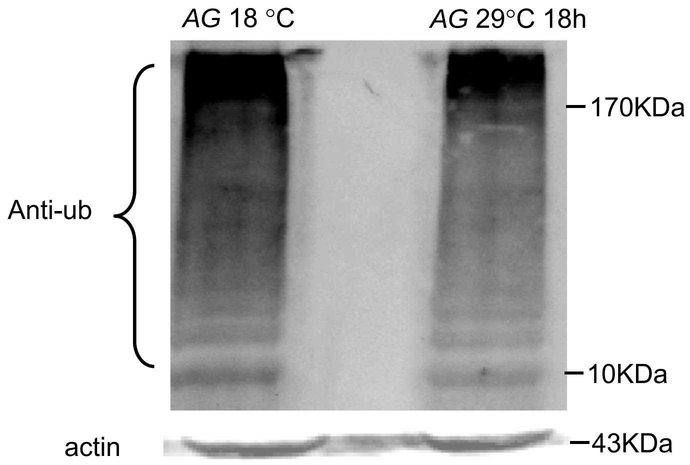

Supplementary Figure 7

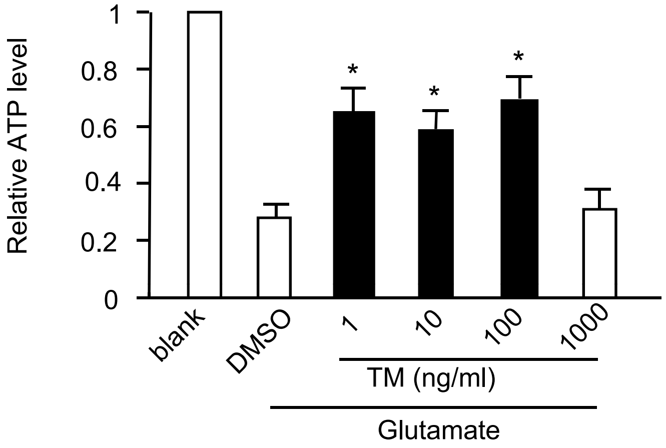

Supplementary Figure 8

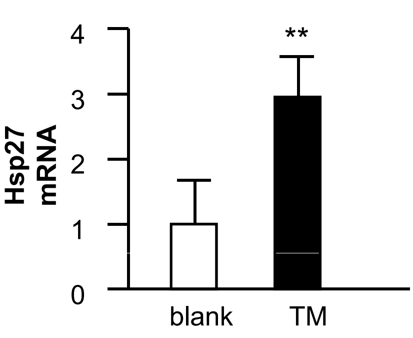

Supplementary Figure 9

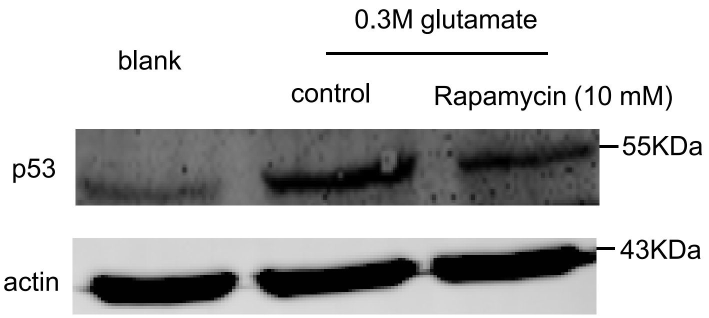

Supplementary Figure 10

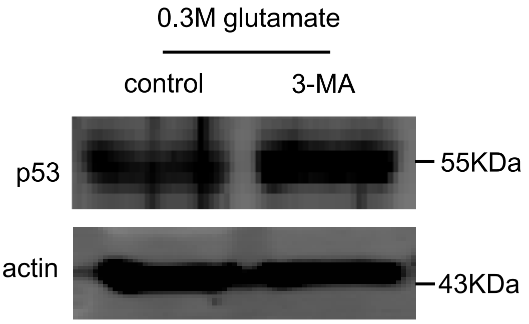

Supplementary Figure 11

Full-length blots

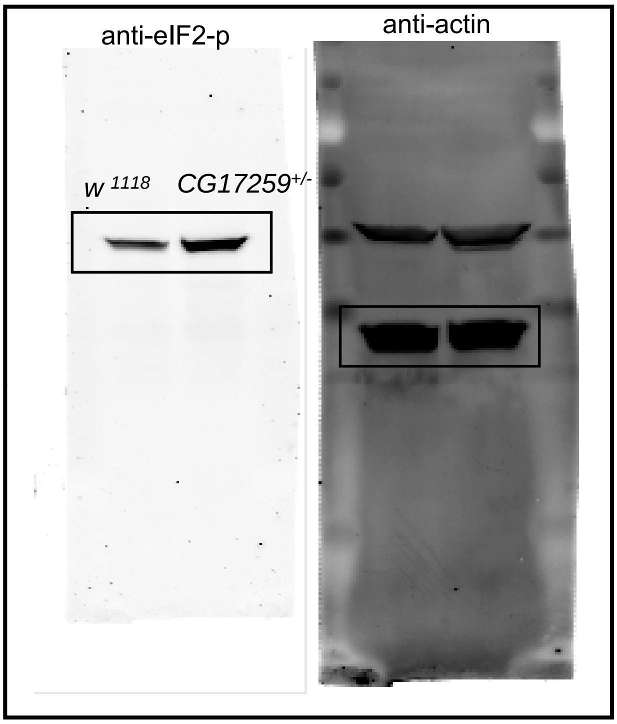

Figure 2b

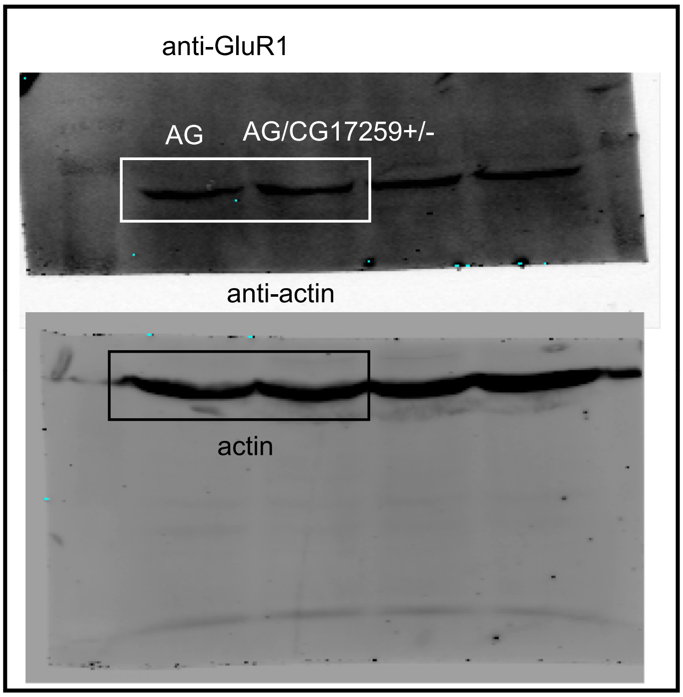

Figure 2c

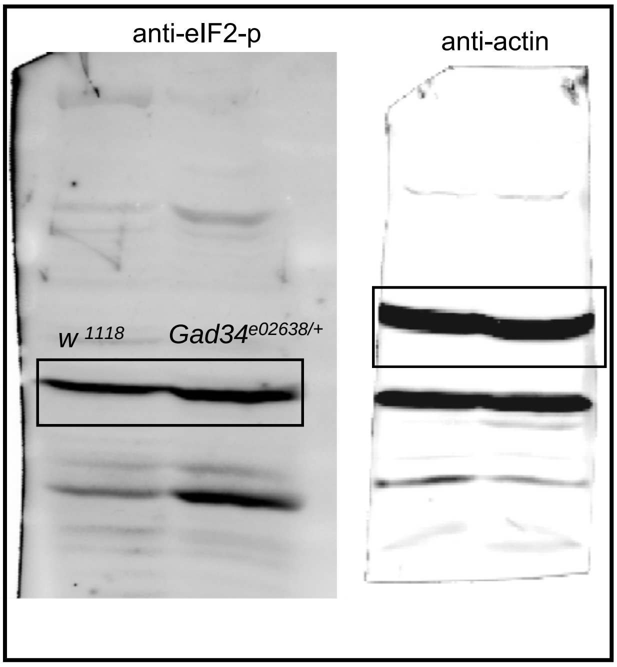

Figure 2d

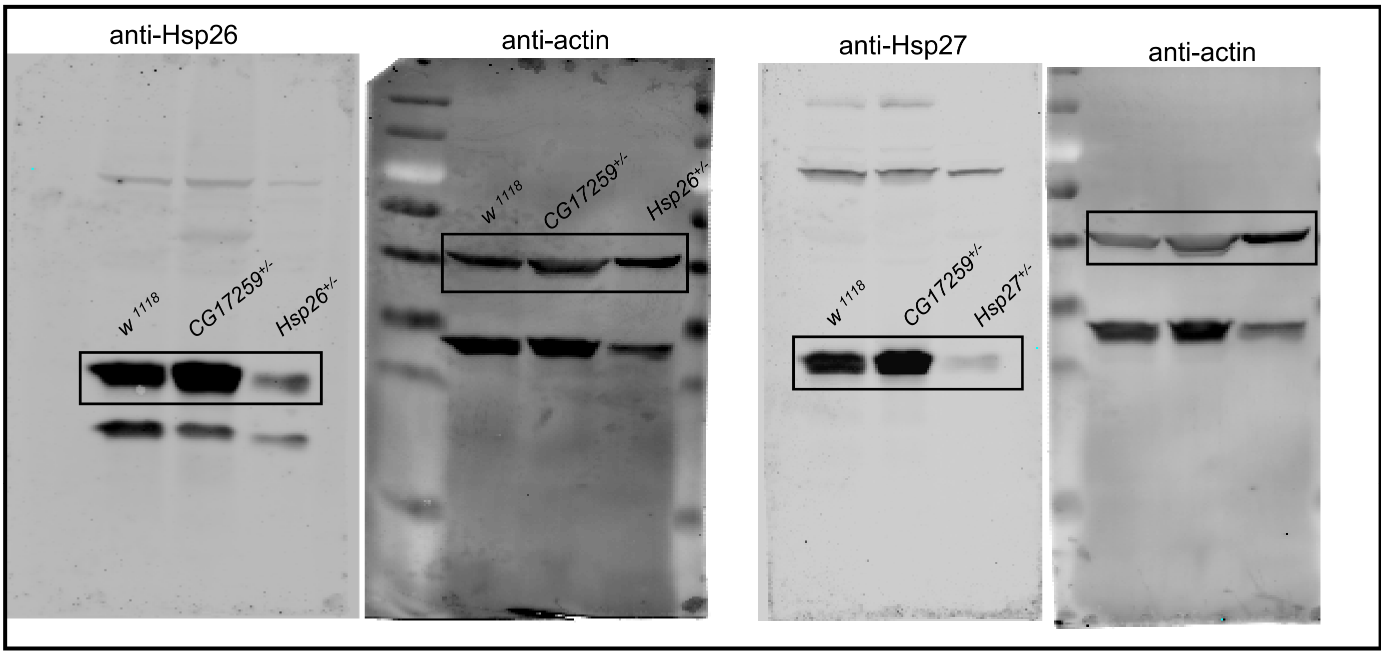

Figure 3b

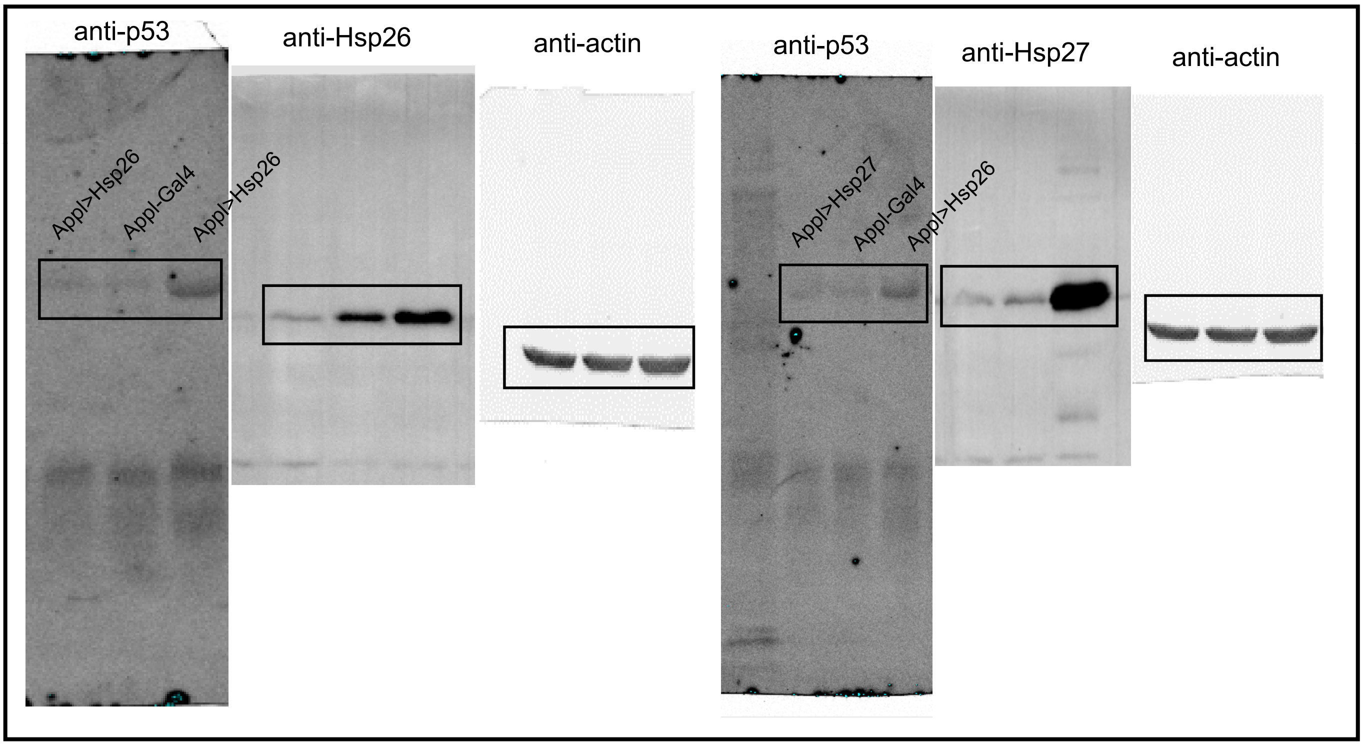

Figure 4a

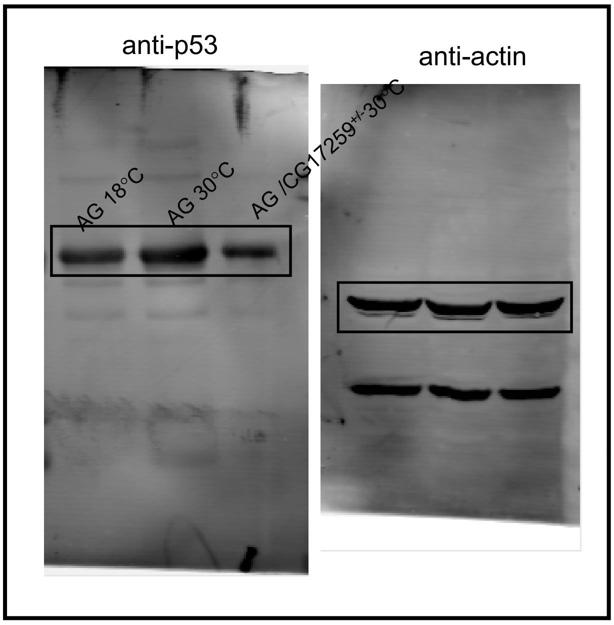

Figure 4b

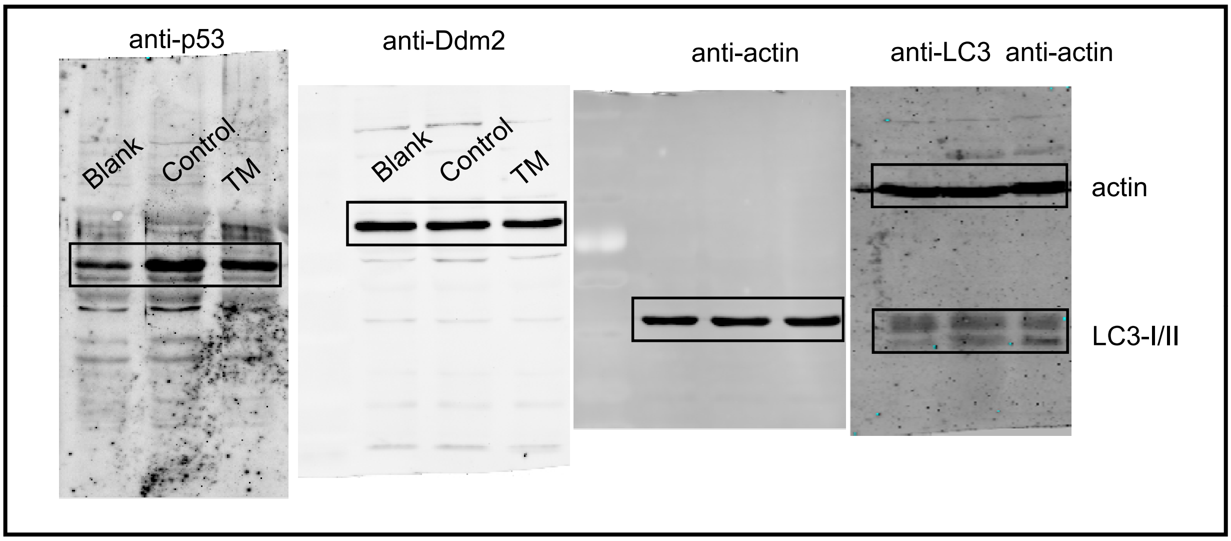

Figure 5c

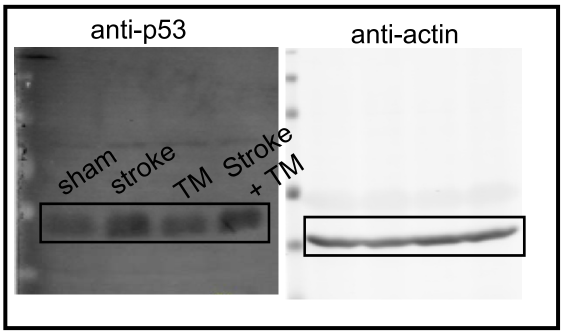

Figure 5f

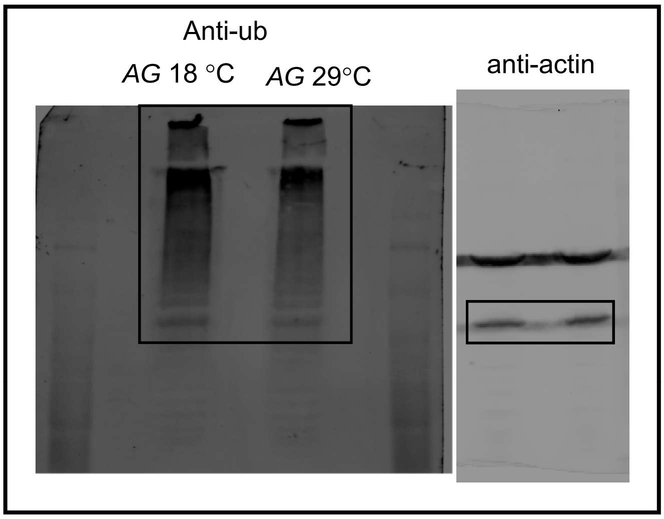

Figure S7

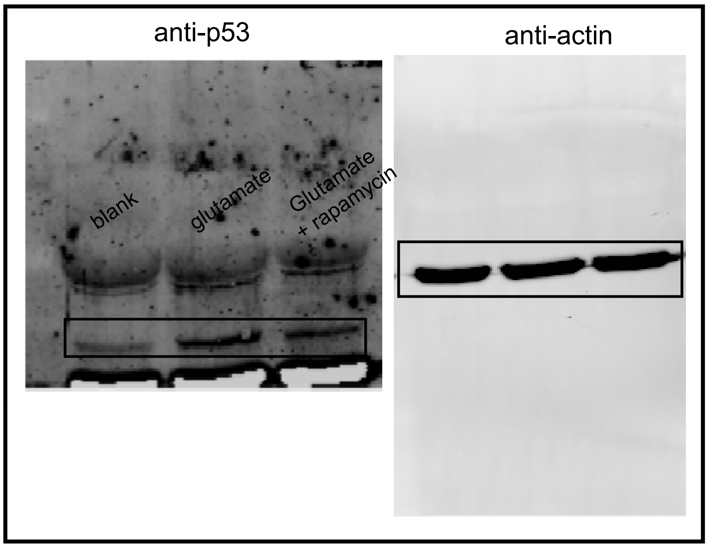

Figure S10

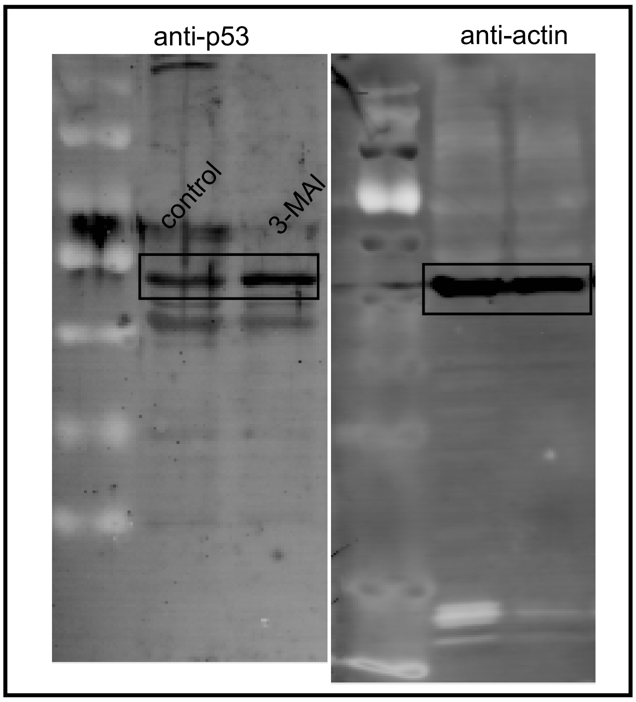

Figure S11
